# Postharvest starch and sugars adjustment in potato tubers of wide-ranging dormancy genotypes subjected to various sprout forcing techniques

**DOI:** 10.1038/s41598-023-37711-y

**Published:** 2023-09-08

**Authors:** Muhammad Wasim Haider, Muhammad Nafees, Rashid Iqbal, Habat Ullah Asad, Farrukh Azeem, Baber Ali, Ghazala Shaheen, Javed Iqbal, Shweta Vyas, Muhammad Arslan, Muhammad Habib Ur Rahman, Mohamed S. Elshikh, M. Ajmal Ali

**Affiliations:** 1 Department of Horticultural Sciences, The Islamia University of Bahawalpur, Bahawalpur, 63100 Pakistan; 2 Department of Agronomy, The Islamia University of Bahawalpur, Bahawalpur, 63100 Pakistan; 3 Centre for Agriculture and Bioscience International, Rawalpindi, 46300 Pakistan; 4 Agri Development, Fauji Fresh N Freeze Ltd, Gulberg II, Lahore, 48000 Pakistan; 5 Department of Plant Sciences, Quaid-I-Azam University, Islamabad, 45320 Pakistan; 6 Department of Eastern Medicine, Faculty of Medicine and Allied Health Sciences, The Islamia University of Bahawalpur, Bahawalpur, Pakistan; 7 Department of Agricultural Engineering, Khwaja Fareed University of Engineering and Information Technology, Rahim Yar Khan, 64200 Pakistan; 8 Department of Pure and Applied Chemistry, University of Kota, Kota, Rajasthan 324001 India; 9 Institute of Crop Science and Resource Conservation (INRES), Crop Science, University of Bonn, 53115 Bonn, Germany; 10 Department of Botany and Microbiology, College of Science, King Saud University, Riyadh, 11451 Saudi Arabia

**Keywords:** Plant physiology, Plant sciences, Ecology

## Abstract

The development of an efficient, safe, and environment-friendly technique to terminate tuber dormancy in potatoes (*Solanum tuberosum* L.) is of great concern due to the immense scope of multiple cropping all over the globe. The breakage of tuber dormancy has been associated with numerous physiological changes, including a decline in the level of starch and an increase in the levels of sugars during storage of freshly harvested seed potatoes, although their consistency across genotypes and various dormancy-breaking techniques have not yet been fully elucidated. The purpose of the present research is to assess the efficacy of four different dormancy-breaking techniques, such as soaking in 90, 60, or 30 mg L^−1^ solutions of benzyl amino purine (BAP) and 30, 20, or 10 mg L^−1^ gibberellic acid (GA3) alone and in the combination of optimized concentrations; cold pre-treatment at 6, 4, or 2 °C; electric shock at 80, 60, 40, or 20 Vs; and irradiation at 3.5, 3, 2.5, 2, 1.5, or 1 kGy on the tuber dormancy period and sprout length of six genotypes. Furthermore, the changes that occurred in tuber weight and endogenous starch, sucrose, fructose, and glucose contents in experimental genotypes following the application of these techniques were also examined. Overall, the most effective technique to terminate tuber dormancy and hasten spout growth was the combined application of BAP and GA_3_, which reduced the length of dormancy by 9.6 days compared to the untreated control, following 6.7 days of electric current, 4.4 days of cold pre-treatment, and finally irradiation (3.3 days). The 60 mg L^−1^ solution of BAP greatly reduced the dormancy period in all genotypes but did not affect the sprout length at all. The genotypes showed a weak negative correlation (r =  − 0.4) (*P* < 0.05) of endogenous starch contents with dormancy breakage and weight loss or a moderate (r =  − 0.5) correlation with sprout length, but a strong positive correlation (r = 0.8) of tuber glucose, fructose, and sucrose contents with dormancy breakage and weight loss. During 3 weeks of storage, sprouting commencement and significant weight loss occurred as tuber dormancy advanced towards breakage due to a reduction in starch and an increase in the sucrose, fructose, and glucose contents of the tubers. These findings could be advantageous for postponing or accelerating seed potato storage as well as investigating related physiological research in the future.

## Introduction

Potato stands first among non-grain food crops and fourth among grain food crops, all over the world, in acreage as well as production^1^. Europe, North America, South America, Asia, and Africa rank among the top five global potato-growing regions. Due to its short growing period, potato offers a quicker crop than cereals or legumes and also produce more food calories per unit by using less water compared to either rice or wheat^2^. In Pakistan, potato is grown on approximately 185.4 thousand hectares with an approximate yearly production of 4552.7 thousand metric tons at an average of 24.6 tons per hectare, a comparatively low average yield^3,4^. The low yield is due to the low quality of the seed^5^. One of the major reasons for decline in quality of seed in Pakistan is the long-term storage of seed potatoes^6,7^, which depends on the autumn-to-autumn cycle for the seed^8^.

Potato is cultivated throughout the year in Pakistan. The main crop is the autumn crop (mid-Sep to mid-Dec) and accounts for 80–85% of the entire production, followed by the spring (1st Jan to 1st April) and summer (1st May to mid-Aug) crops, accounting for 10–15% and 1–2% of the production, respectively^9^. But its production has been hindered by seed storage and dormancy issues^10^. The lack of suitable storage facilities has contributed to a significant seed loss and reduced crop quality. Furthermore, storing seed potatoes for extended periods poses a challenge due to their tendency to undergo dormancy. Dormancy after harvesting hinders the sprouting of the tubers, even under promising ecological conditions leading to reduced sprouting and compromised yields^11^. Moreover, the spring and summer harvests cannot be utilized as seed for the next summer and autumn crops. Likewise, the seed harvested in the autumn could not be sown for the next spring and summer crops. The economic period of dormancy differs from country to country, depending on the cropping pattern. The time between harvesting and sowing subsequent crops is very short (< 1 month) in Pakistan, thereby diminishing the scope of multiple cropping. The availability of short-term dormancy genotypes and effective and safe-to-use dormancy breaking technique can enable growers to get two or three crops a year. As seed tubers are living entities and respire during storage, they lose their weight and hence their quality^12^. The percentage of tuber weight loss is considerably genotype- and storage-environment-dependent^13,14^. Tubers with sprouts lose more weight compared to unsprouted ones since there is a significant relationship between sprout growth and weight loss^15^. The quality of potato tubers under storage conditions can be influenced by various factors such as temperature, humidity, storage duration, and pre-storage treatments^16,17^. Potato comprises approximately 68% starch of the total tuber dry matter, which indicates a high demand for carbohydrates^18^. The contents of tuber starch, sucrose, fructose, and glucose substantially depend on growth and development stage^19^. During tuberization, a massive buildup of starch occurs in the tubers with the highest sucrose, fructose, and glucose contents^20^, and as they mature physiologically, these contents tend to decline^21^. According to previous studies, immature tubers contain 0.2–1.5% sucrose and 0.01–0.7% reducing sugars^22^, while mature tubers contain 0.1–0.6% sucrose and 0.04–0.4% reducing sugars^21,23^. A rapid accumulation of soluble sugars occurs during instinctive dormancy breakage^24,25^. Although their exact levels in genotypes are still unknown.

The dormancy breakage can also be governed by various dormancy breaking approaches^26^. The most popular techniques include the use of various chemicals such as carbon disulfide^27^, bromoethane^28^, rindite^29^, and thiourea^30^. However, they are either inefficient or harmful to both people and the environment. Ecofriendly and harmless techniques are always preferred for sustainable agriculture. The application of plant growth regulators (PGRs) in low quantities, cold pre-treatment, electric current, and irradiation are reported to terminate tuber dormancy and stimulate sprouting in potatoes and are also safe to use for humans. Combined applications of BAP and GA_3_ could be advantageous, as BAP, being a cytokinin, terminates the tuber dormancy by attracting assimilates, while GA_3_ is responsible for mobilizing assimilates for sprout growth^31^. Electric current breaks tuber dormancy by inducing GA_3_ production when employed at a certain voltage^10,32^. Cold shock and γ-rays are known to affect the sugar levels of potato tubers. The reducing and non-reducing sugars both inevitably rose within a week of exposure to radiations as earlier reported by Haider et al.^10^ and Amjad et al.^33^ which were then utilized by the growing sprout. Although the exact levels of starch and sugars at dormancy breakage in response to various dormancy breaking techniques are undefined yet. Understanding these endogenous changes in the tuber may help to choose a suitable dormancy-breaking technique based on available resources to force the sprouting of potatoes for their use as seed. In this study, we aim to investigate the efficacy of four different methods for breaking seed tuber dormancy in potatoes. By exploring these methods, we seek to provide valuable insights and potential solutions to enhance seed tuber quality for improved potato yields.

## Materials and methods

### Plant materials

**Figure 1:**
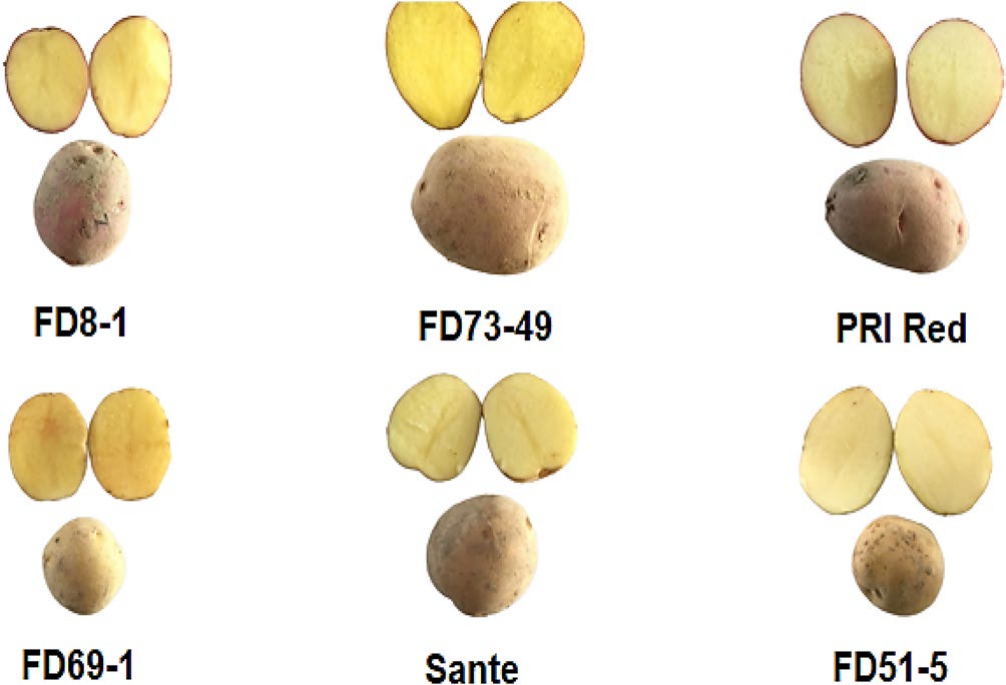
Selected red and white skin potato genotypes varying in dormancy period.

Six potato (*Solanum tuberosum* L.) genotypes screened out of 22 containing three red (FD8-1, FD73-49, and PRI Red) and white skin genotypes (FD69-1, Sante, and FD51-5) (Fig. 1) were acquired from the Potato Research Institute (PRI) (30°^nn^67′ N and 73° 16′ E with an altitude of 152 m), Sahiwal, Pakistan^6^. Based on their dormancy behaviour, only healthy tubers were chosen from each genotype for the study of dormancy period, sprout length, weight loss, starch, sucrose, fructose, and glucose contents, excluding damaged, diseased, or misshapen tubers, after application of various dormancy breaking techniques given in detail below.

### Application of dormancy breaking techniques

During March 2018, tubers from the PRI, Sahiwal were collected 10 days after harvesting. Tubers were immersed in benzylaminopurine (BAP) solutions of 90, 60, or 30 mg L^−1^ and gibberellic acid (GA_3_) solutions of 30, 20, or 10 mg L^−1^ for 24 h in order to optimize their concentrations. The tubers were then immersed in the PGRs’ solutions at their optimized concentrations to evaluate their cumulative effect on dormancy breakage and subsequent sprouting. Tubers were immersed in distilled water as a control. Each treatment was repeated three times, and 30 tubers were utilized in every replication. The parenchyma tissues of potatoes were exposed to PGRs’ solutions by making a tiny cut (10 mm depth × 15 mm length) due to the impermeability of potato skin to chemicals^10^. After treatment, the tubers were kept at room temperature (23.8 ± 1.0 °C) until sprouting.

Secondly, tubers were packed in perforated cardboard boxes and transported to the Ayyub Agricultural Research Institute's Postharvest Research Center for low-temperature treatment. The tubers were kept in storage for four days under various low temperature conditions, including 6 °C, 4 °C, 2 °C, and control.

Tubers were subjected to electric current (80 V, 60 V, 40 V, or 20 V) for 24 h by having needles (of a hand-made electric stimulator) inserted 15 mm into the flesh at the tubers’ stem and apical ends. In control tubers, needles were injected but without electric current. After being treated, the tubers were kept at room temperature.

For evaluation of the impact of γ-rays on tuber starch and sugar contents, a 137Cs source with a radiation output of 1 kGy/1.5 h was utilized at the Nuclear Institute of Agriculture and Biology (NIAB) in Faisalabad.

All the above experiments were arranged according to a completely randomized design (CRD) in factorial settings.

### Data collection

#### Measurement of dormancy duration and sprout length

Six tubers in each replication were used to measure the dormancy time and sprout length on a daily basis. The dormancy was considered broken when the tubers' sprouts gained a length of 2 mm^10,15,34^. The length of the sprout was taken with a measuring scale.

#### Determination of tuber weight loss

Tuber weight loss was calculated by the formula given below. In which “T_0_” represents the initial weight of tubers following the harvest, while “T” represents the final weight of tubers following 3-week storage. The final values were calculated by taking the mean of three replicates. The selected tubers were labeled for each genotype and weighed using an electronic balance (DM-01, ScaleTech, Beijing, China).

$${\text{Tuber weight loss }}(\% ) = \frac{{T_{{\text{o}}} - T}}{{T_{{\text{o}}} }} \times 100$$

#### Determination of starch contents in tubers

Starch estimation was carried out by anthrone reagent^35^. A fresh sample weighing 0.5 g was homogenized in hot 80% ethanol and centrifuged at 10,000 rpm for 20 min. The residue was then mixed with 5 mL of water and 6.5 mL of perchloric acid, and it was then refrigerated for 20 min at 2 °C. The leftover material was again centrifuged, and the supernatant was saved for examination. The final volume was increased to 100 mL, and distilled water was used to dilute it in a ratio of 1:5. Each test tube received 4 mL of the anthrone reagent, which was then heated in a boiling water bath for around 8 min. The content was rapidly cooled. A UV–Vis spectrophotometer (2326 K, Hermle Labortechnik GmbH, Wehingen, Germany) was used to measure the absorbance of the collected supernatant at 630 nm. The starch contents of the fresh potato samples were determined using a series of glucose working standards solutions (20–100 µg mL^−1^).

#### Quantification of endogenous sugar contents in tubers

Sugar levels were quantified using HPLC 1 week and 3 weeks after the treatment of tubers^36^. A 20 g sample of the tuber was extracted for 10 min in 40 mL of distilled water with a magnetic stirrer to dissolve the sugars. After that, the extracts were centrifuged for 10 min at 13,000×*g* to separate the supernatants. Each sample was filtered through a 0.45 μm membrane filter prior to HPLC analysis.

#### Settings for liquid chromatography (LC)

LC separation was carried out at room temperature on a Razex RCM-Monosaccharidses Ca^2+^—Phenomenex. The mobile phase was 100% double-distilled water. HPLC was connected to a refractive index detector (ReID) RID-10 AL (Shimadzu, Japan). The column temperature was 25 °C. The injection volume and flow rate were 20 μL and 1 mL min^−1^, respectively. Detected quantities of sugars were determined from peak areas of external standards consisting of sucrose (1%), fructose (1%), and glucose (1%) solutions^37^. Results were expressed as a percentage of dry weight.

### Statistical analyses

All data were subjected to a three-way analysis of variance (genotype, treatment, and storage period) using Statistix9^®^ software (Analytical Software, Tallahassee, USA). Results are interpreted as the relative contribution of genotype, treatment, storage period, and their interactions by calculating the percentage of total variance from the corresponding sum of squares^38^. For mean comparisons of main effects for genotype, treatment, storage period, and their interactions at *P* ≤ 0.05, the least significant difference (LSD) test was applied. The Pearson's correlation analysis was performed by the general linear model procedure in SAS, version 9.2 (Cary, NC).

### Ethical approval

It is stated that the research complies with relevant institutional, national, and international guidelines and legislation.

## Results

### Screening trial

There were significant differences in the post-harvest dormancy period among the original 22 genotypes evaluated in the screening trial^6^. From these, three distinct groups were chosen based on their dormancy behavior. Short-term dormancy genotypes included PRI Red and FD51-5; medium-term dormancy genotypes included FD73-49 and Sante; and FD8-1 and FD69-1 were classified as long-term dormancy genotypes. Each group contained one red and one white skin genotype since consumers in the country (Pakistan) prefer and consume these two colors of tubers equally.

### Individual application of PGRs and their optimization

#### Effect on tuber dormancy period, sprout length and weight loss

PGRs, genotypes, storage periods, and their interactions had a significant (*P* ≤ 0.05) effect on tuber dormancy period, sprout length, and weight loss (Table 1). Among PGRs, 60 mg L^−1^ solution of BAP shortened the tuber dormancy period most effectively in all genotypes, whereas 20 mg L^−1^ of GA_3_ was the most effective in increasing sprout length(Table 1). The highest weight loss (2.17%) was also noted in the tubers soaked in a 60 mg L^−1^ solution of BAP, followed by those soaked in a 20 mg L^−1^ of GA_3_. Among genotypes, PRI Red exhibited the shortest dormancy period (13.7 days) (Table 1), and FD8-1 took the longest period (30.9 days) to dormancy breakage. With the increase in storage period, the dormancy period reduced, and sprout growth and weight loss increased (Table 1).

**Table 1:** Effect of PGRs on tuber dormancy period, sprout length and weight loss of six potato genotypes in relation to endogenous changes occurred in starch, sucrose, fructose and glucose contents of tuber.

Factors	DP (days)	SL (mm)	WL (%)	Starch (%)	Sucrose (%)	Fructose (%)	Glucose (%)
Treatment (T) (mg L^−1^)							
Control	27.9a	1.36f	1.95e	11.76a	0.80e	0.214d	0.741d
BAP 90	21.3d	1.64d	2.03c	11.59b	0.86b	0.228b	0.780b
BAP 60	18.4f	1.76c	2.17a	11.07e	0.93a	0.257a	0.864a
BAP 30	24.5b	1.54e	1.98d	11.49c	0.82cde	0.216cd	0.764c
GA_3_ 30	22.9c	1.82c	0.88d	11.38d	0.81de	0.215d	0.746d
GA_3_ 20	20.3e	2.05a	2.07b	10.91f	0.83c	0.220c	0.763c
GA_3_ 10	22.8c	1.92b	2.01c	11.50c	0.83cd	0.221c	0.766bc
LSD T (P ≤ 0.05)	0.58	0.082	0.022	0.064	0.016	0.004	0.014
Genotype (G)							
FD8-1	30.9a	0.9e	1.71e	14.92b	0.87d	0.21d	0.82d
FD69-1	29.6b	1.1d	1.79d	14.68a	0.92c	0.23c	0.84c
FD73-49	21.5d	1.7b	2.15b	11.45c	0.50f	0.12f	0.45f
Sante	22.6c	1.6c	2.10c	10.71d	1.01b	0.29b	0.92b
PRI Red	13.7f	3.4a	2.23a	7.81f	1.08a	0.32a	1.00a
FD51-5	17.1e	1.7bc	2.15b	8.73e	0.65e	0.14e	0.59e
LSD G (P ≤ 0.05)	0.53	0.077	0.020	0.060	0.015	0.004	0.013
Storage period (SP)							
Week 1	0.0b	0.00b	0.00b	12.17a	0.35b	0.09b	0.32b
Week 3	45.1a	3.47a	4.05a	10.61b	1.33a	0.35a	1.22a
LSD SP (P ≤ 0.05)	0.31	0.044	0.012	0.034	0.008	0.002	0.007
LSD T × G (P ≤ 0.05)	1.41	0.203	0.053	0.159	NS	NS	NS
LSD T × SP (P ≤ 0.05)	0.82	0.117	0.031	0.056	0.023	0.006	0.020
LSD G × SP (P ≤ 0.05)	0.76	0.108	0.029	0.051	0.021	0.006	0.019
LSD T × G × SP (P ≤ 0.05)	1.99	0.287	0.075	0.225	NS	NS	NS

*NS* non-significant at *P* ≤ 0.05. Treatment means sharing the same letter are non-significantly different. LSD is the least significant difference.

*DP* dormancy period, *SL* sprout length, *WL* weight loss.

**Figure 2:**
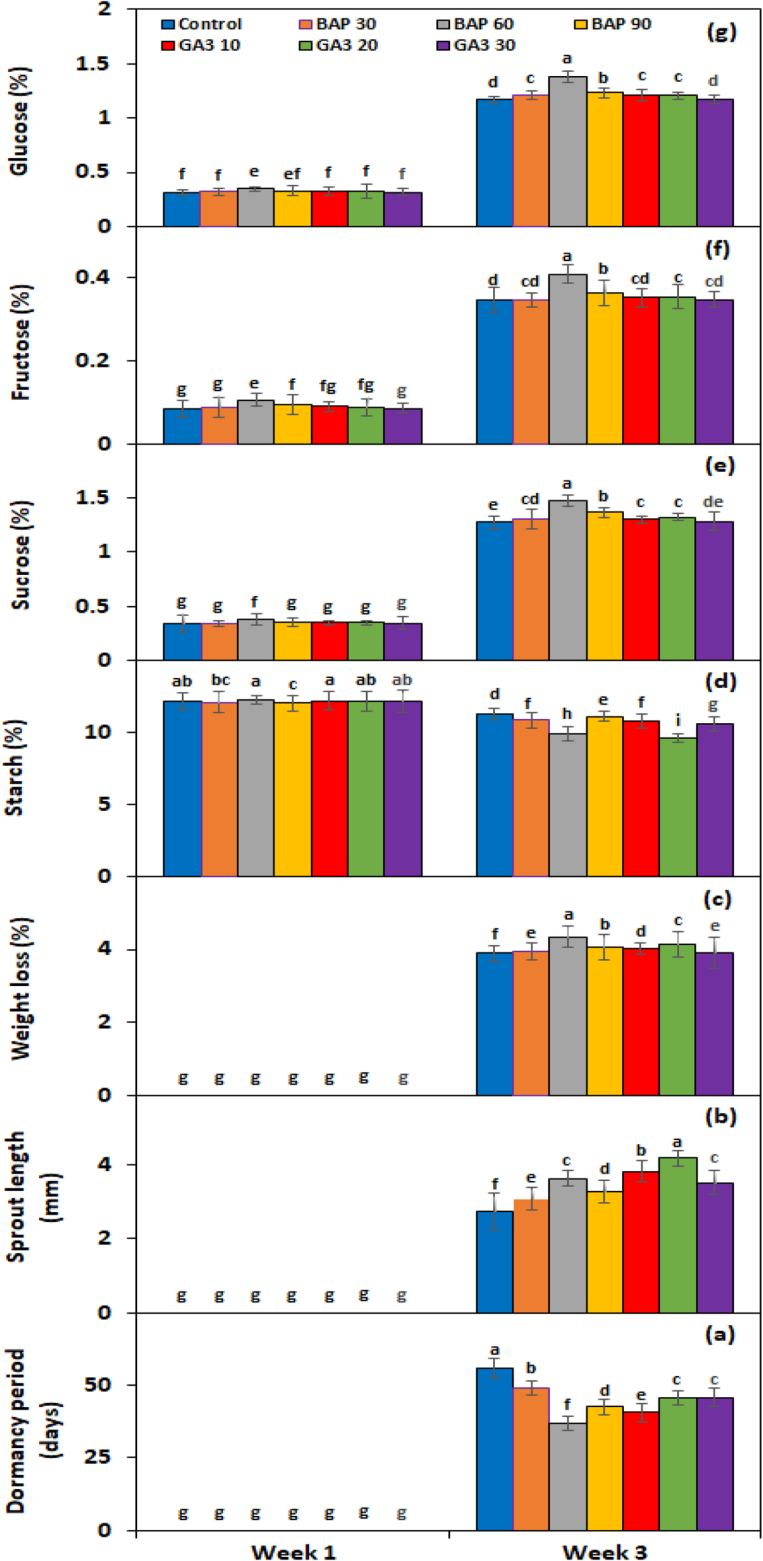
Interactive effect of *treatment* × *storage period* on tuber dormancy period (**a**), sprout length (**b**), weight loss (**c**), starch (**d**), sucrose (**e**), fructose (**f**), and glucose contents (**g**) of six potato genotypes subjected to BAP and GA_3_ solutions. The treatment means sharing the same letter are non-significant (*P* > 0.05) according to the least significant difference test. The vertical bars represent the standard error of means (n: 3).

**Figure 3:**
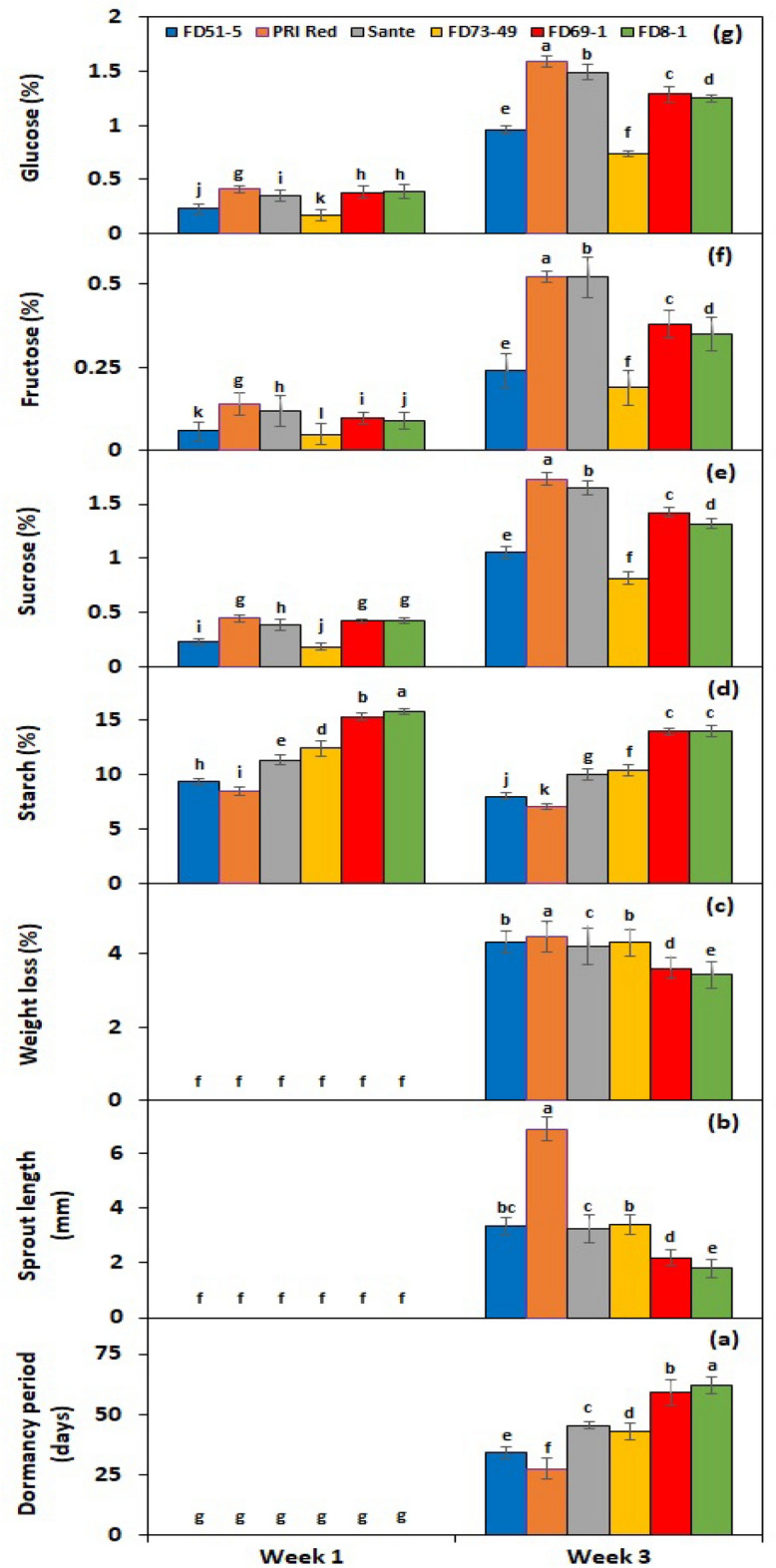
Interactive effect of *genotype* × *storage period* on tuber dormancy period (**a**), sprout length (**b**), weight loss (**c**), starch (**d**), sucrose (**e**), fructose (**f**), and glucose contents (**g**) of six potato genotypes subjected to BAP and GA_3_ solutions. The treatment means sharing the same letter are non-significant (*P* > 0.05) according to the least significant difference test. The vertical bars represent the standard error of means (n: 3).

Under the *PGRs* × *storage period* interaction, BAP showed a significant effect on all experimental genotypes after the third week of storage in terms of dormancy breakage (Fig. 2a), while GA_3_ in terms of sprout growth (Fig. 2b). Whereas weight loss was observed at its maximum (Fig. 2c) in the tubers stored for 3 weeks after soaking in 60 mg L^−1^ BAP. Under *genotype* × *storage period* interaction, PRI Red advanced rapidly towards dormancy breakage (27.3 days) (Fig. 3a), with the longest sprout at week 3 (6.9 mm) (Fig. 3b), due to which it remarkably dropped its weight (4.5%) (Fig. 3c). On the other hand, FD8-1 displayed the longest tuber dormancy period (61.7 days) (Fig. 3a), with the shortest sprout length (1.7 mm) (Fig. 3b), and the lowest weight loss (3.4%) (Fig. 3c).

#### Effect on endogenous starch, sucrose, fructose and glucose contents of tuber

The starch, sucrose, fructose, and glucose contents in the experimental tubers were significantly affected by PGRs, genotypes, storage periods, and the two-way interactions of *PGRs* × *storage period* and *genotype* × *storage period* (Table 1). The starch contents were significantly decreased (10.91%) in the tubers treated with a 20 mg L^−1^ solution of GA_3_, followed by 60 mg L^−1^ of BAP (11.07%) (Table 1). On the other hand, the sucrose (0.93%), fructose (0.257%), and glucose (0.864%) contents of tubers were found highest in the tubers treated with 60 mg L^−1^ of BAP, followed by 90 mg L^−1^ of BAP. Among GA_3_ levels, the treatment with 20 mg L^−1^ developed the highest contents of sucrose, fructose, and glucose. Among genotypes, FD69-1 exhibited the highest level (14.68%) of starch, while PRI depicted the lowest level (7.81%) (Table 1). Alternatively, sucrose, fructose, and glucose were found to be highest in PRI Red and lowest in FD73-49 (Table 1). There was a weak negative correlation (*r* =  − 0.4) (*P* < 0.05) of starch contents with tuber dormancy period and weight loss or a moderate (*r* =  − 0.5) correlation with sprout length. However, tuber glucose, fructose, and sucrose contents had a strong positive correlation (*r* ≥ 0.8) with dormancy period, sprout length, and weight loss. From the first to the third week of storage, starch contents declined while sucrose, fructose, and glucose contents elevated (Table 1).

Under *PGRs* × *storage period* interaction, BAP and GA_3_ showed a comparatively greater effect on tuber starch (Fig. 2d), sucrose (Fig. 2e), fructose (Fig. 2f), and glucose (Fig. 2g) contents than the untreated control. Under genotype × storage period interaction, starch contents decreased more rapidly in the short-term dormancy genotypes than in the moderate or long-term dormancy genotypes (Fig. 3d). On the other hand, sucrose (Fig. 3e), fructose (Fig. 3f), and glucose (Fig. 3g) contents were developed in small quantities in the tubers of PRI Red during the first week in comparison with the contents during the third week.

### Individual and combined application of optimized levels of PGRs

#### Effect on tuber dormancy period, sprout length and weight loss

The main effects of PGRs, genotypes, and storage periods, as well as their interactive effects, had a significant (*P* ≤ 0.05) influence on tuber dormancy period, sprout length, and weight loss (Table 2). Among PGRs, the combination of 60 mg L^−1^ BAP with 20 mg L^−1^ GA_3_ efficiently broke the dormancy of tubers within 18.1 days and increased the sprout length by 33.6% (Table 2). The tubers soaked in both 60 mg L^−1^ BAP and 20 mg L^−1^ GA_3_ solutions also demonstrated the highest weight loss (2.31%). Among genotypes, PRI Red exhibited the shortest dormancy period (13.8 days) with the highest sprout length (3.5 mm) and weight loss (2.27%), whereas FD8-1 took the longest period (30.6 days) to dormancy breakage with the smallest sprout length (0.95 mm) and weight loss (1.79%) (Table 2). With the increase in storage period, dormancy decreased and sprout growth and weight loss increased (Table 2).

**Table 2:** Individual and combined effect of optimized PGRs levels on tuber dormancy period, sprout length and weight loss of six potato genotypes and relative changes occurred in endogenous starch, sucrose, fructose and glucose contents.

Factors	DP (days)	SL (mm)	WL (%)	Starch (%)	Sucrose (%)	Fructose (%)	Glucose (%)
Treatment (T) (mg L^−1^)							
Control	27.7a	1.36c	1.90d	11.62a	0.81d	0.20d	0.73d
BAP 60	18.7c	1.71b	2.04c	10.94b	0.96b	0.25b	0.86b
GA3 20	23.3b	2.01a	2.16b	10.76c	0.84c	0.21c	0.76c
BAP 60 + GA3 20	18.0c	2.08a	2.31a	10.58d	0.99a	0.25a	0.89a
LSD T (P ≤ 0.05)	0.75	0.076	0.017	0.068	0.018	0.004	0.017
Genotype (G)							
FD8-1	30.6a	0.95e	1.79d	14.63a	0.96c	0.22d	0.87c
FD69-1	28.8b	1.14d	1.87c	14.30b	0.97c	0.24c	0.87c
FD73-49	21.5c	1.75b	2.25a	10.88c	0.54e	0.12f.	0.49e
Sante	21.6c	1.65c	2.17b	10.40d	1.06b	0.30b	0.96b
PRI Red	13.8e	3.55a	2.27a	7.26f.	1.15a	0.33a	1.05a
FD51-5	15.4d	1.65c	2.27a	8.37e	0.72d	0.16e	0.64d
LSD G (P ≤ 0.05)	0.91	0.093	0.021	0.083	0.02	0.005	0.021
Storage period (SP)							
Week 1	0.0b	0.0b	0.00b	12.05a	0.36b	0.09b	0.33b
Week 3	43.9a	3.57a	4.21a	9.89b	1.44a	0.37a	1.30a
LSD SP (P ≤ 0.05)	0.53	0.05	0.006	0.009	0.013	0.003	0.012
LSD T × G (P ≤ 0.05)	1.83	0.19	0.043	0.167	NS	NS	NS
LSD T × SP (P ≤ 0.05)	1.05	0.11	0.025	0.096	0.026	0.006	0.025
LSD G × SP (P ≤ 0.05)	1.29	0.13	0.030	0.118	0.032	0.007	0.030
LSD T × G × SP (P ≤ 0.05)	2.58	0.26	0.061	0.237	NS	NS	NS

*DP* dormancy period, *SL* sprout length, *WL* weight loss, *BAP* benzylaminopurine, *GA3* gibberellic acid.

*NS* non-significant at *P* ≤ 0.05. Treatment means sharing the same letter are non-significantly different. LSD is the least significant difference.

**Figure 4:**
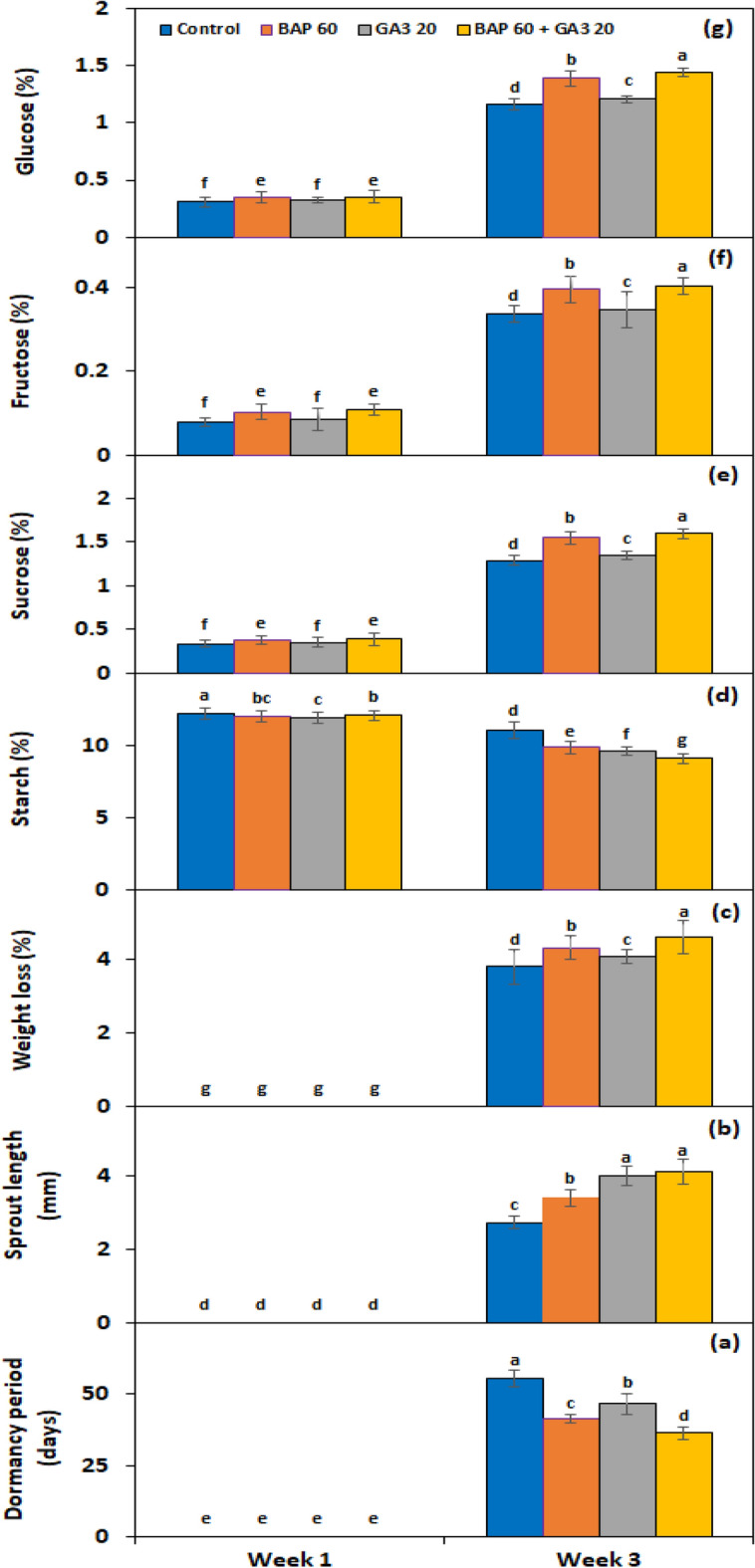
Interactive effect of *treatment* × *storage period* on tuber dormancy period (**a**), sprout length (**b**), weight loss (**c**), starch (**d**), sucrose (**e**), fructose (**f**), and glucose contents (**g**) of six potato genotypes subjected to optimized solutions BAP and GA_3_ alone and in combination. The treatment means sharing the same letter are non-significant (*P* > 0.05) according to the least significant difference test. The vertical bars represent the standard error of means (n: 3).

**Figure 5:**
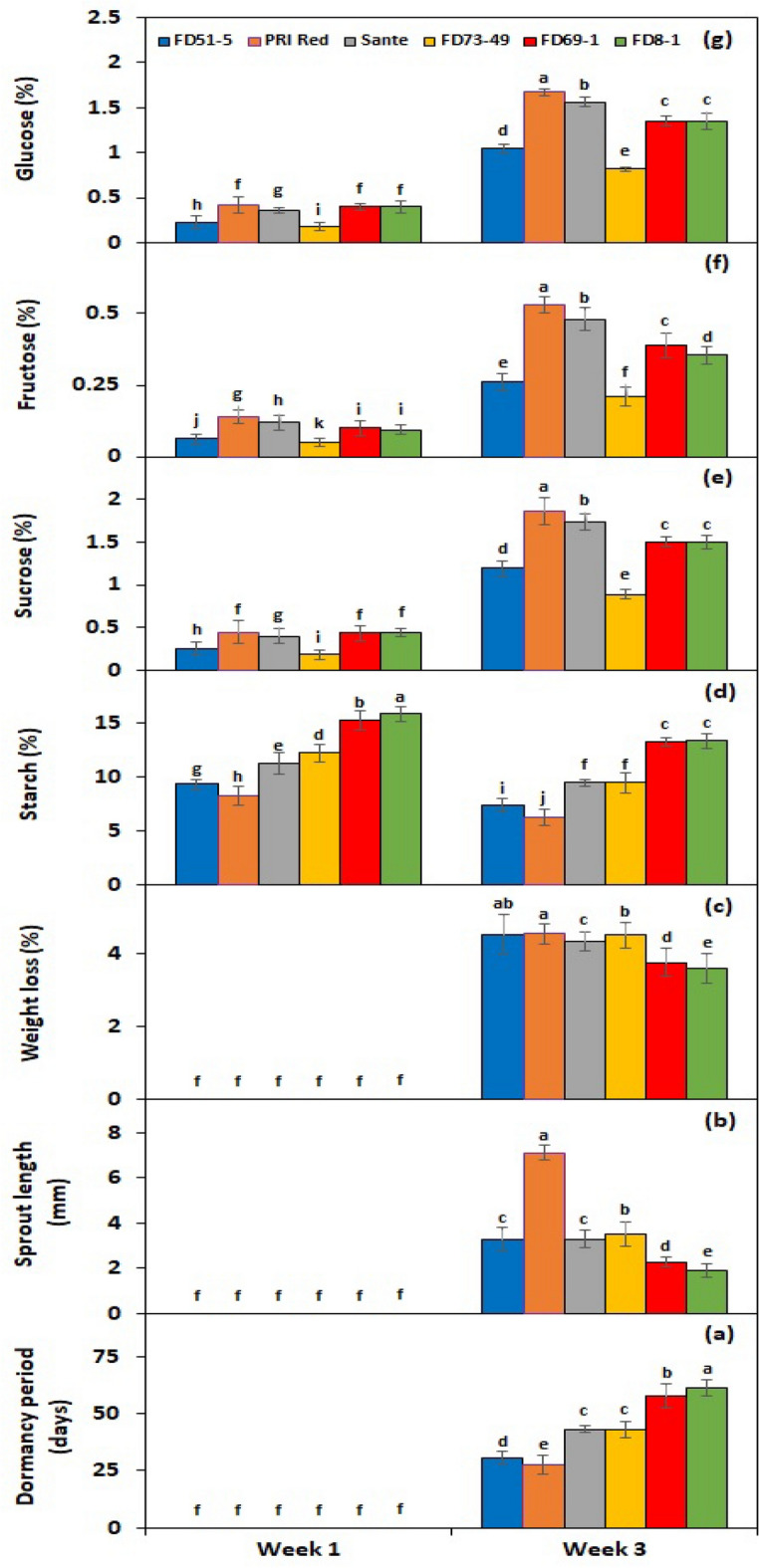
Interactive effect of *genotype* × *storage period* on tuber dormancy period (**a**), sprout length (**b**), weight loss (**c**), starch (**d**), sucrose (**e**), fructose (**f**), and glucose contents (**g**) of six potato genotypes subjected to optimized solutions BAP and GA_3_ alone and in combination. The treatment means sharing the same letter are non-significant (*P* > 0.05) according to the least significant difference test. The vertical bars represent the standard error of means (n: 3).

Under *PGRs* × *storage period* interaction, BAP and GA_3_ showed a significant cumulative effect on all experimental genotypes after the third week of storage, with the quickest dormancy breakage (Fig. 4a) and the greatest sprout length (Fig. 4b); as a result, the highest weight loss (Fig. 4c). Under *genotype* × *storage period* interaction, PRI Red advanced rapidly towards dormancy breakage (27.8 days) (Fig. 5a), with the longest sprout at week 3 (7.3 mm) (Fig. 5b) due to the rapid rate of decrease in starch contents and increase in sugar contents, which resulted in a remarkable drop in its weight (4.6%) (Fig. 5c). On the other hand, FD8-1 displayed the longest tuber dormancy period (61.3 days) (Fig. 5a), the shortest sprout length (1.9 mm) (Fig. 5b), and the lowest weight loss (3.5%) (Fig. 5c).

#### Effect on endogenous starch, sucrose, fructose and glucose contents of tuber

The starch, sucrose, fructose, and glucose contents in the experimental tubers were significantly (*P* ≤ 0.05) influenced by PGRs, genotypes, storage periods, and the two-way interactions of *PGRs* × *storage period* and *genotype* × *storage period* (Table 2). The application of 60 mg L^−1^ of BAP and 20 mg L^−1^ of GA_3_ together resulted in the lowest starch (10.58%) and highest sucrose (0.99%), fructose (0.25%), and glucose (0.89%) levels. The highest starch contents among genotypes were found in FD8-1, while the lowest were found in PRI Red (Table 2). Alternatively, sucrose, fructose, and glucose were found to be highest in PRI Red and lowest in FD73-49 (Table 2). There was a weak negative (*r* =  − 0.1) correlation of starch contents with tuber dormancy period and a moderate to strong (*r* =  − 0.5 to 0.65) correlation with sprout length and weight loss, respectively. However, tuber glucose, fructose, and sucrose contents had a strong positive correlation (*r* ≥ 0.8) with dormancy period, sprout length, and weight loss. During 3 weeks of storage, starch contents reduced while sucrose, fructose, and glucose contents aggravated (Table 2).

Under *PGRs* × *storage period* interaction, BAP and GA_3_ together decreased the starch contents by 61.2% (Fig. 4d) and increased the sucrose (Fig. 4e), fructose (Fig. 4f), and glucose (Fig. 4g) contents by 21%, 13.2%, and 20.9%, respectively. Under the interactive effect of genotype × storage period, starch levels dropped more quickly in the short-term dormancy genotypes than in the moderate or long-term dormancy genotypes (Fig. 5d). On the other hand, sucrose (Fig. 5e), fructose (Fig. 5f), and glucose (Fig. 5g) contents were developed in small quantities in the tubers of PRI Red during the first week in comparison with the contents during the third week.

### Cold pre-treatment of tubers

#### Effect on tuber dormancy period, sprout length and weight loss

The tuber dormancy period, sprout length, and weight loss were significantly (*P* ≤ 0.05) affected by the cold storage temperature, genotypes, storage periods, and their interactions (*temperature* × *storage period* and *genotype* × *storage period*) (Table 3). The tuber weight loss was also affected by *temperature* × *genotype* and *temperature* × *genotype* × *storage period* (Table 3). All other interactions were found to be non-significant (*P* > 0.05) (Table 3). Among storage temperatures, 2 °C resulted in a significant reduction in dormancy period (22.5 days) and an increase in sprout length (1.67 mm) in all genotypes (Table 3). Also, the highest weight loss (2.04%) was recorded in the tubers kept at 2 °C. Among genotypes, PRI Red exhibited the shortest dormancy period (16.3 days), highest sprout length (3.15 mm), and weight loss (2.18%), while FD8-1 exhibited the longest period (33.6 days), smallest sprout length (0.64 mm), and weight loss (1.58%) (Table 3). With the increase in storage period, tuber dormancy reduced, while sprout length and weight loss increased.

**Table 3:** Effect of cold pre-treatment on tuber dormancy period, sprout length and weight loss of six potato genotypes and relative changes occurred in endogenous starch, sucrose, fructose and glucose contents.

Factors	DP (days)	SL	WL (%)	Starch (%)	Sucrose	Fructose (%)	Glucose (%)
		(mm)			(%)		
Temperature (T)							
Control	26.9a	1.37c	1.88c	10.79a	0.80d	0.20b	0.73c
6 °C	25.6b	1.38c	1.83d	10.53ab	0.82c	0.21b	0.74c
4 °C	24.3c	1.50b	1.95b	10.43b	0.83b	0.21b	0.76b
2 °C	22.5d	1.67a	2.04a	10.27b	0.88a	0.23a	0.79a
LSD T (P ≤ 0.05)	0.84	0.098	0.036	0.341	0.012	0.004	0.011
Genotype (G)							
FD8-1	33.6a	0.64d	1.58e	14.51a	0.90c	0.21d	0.83c
FD69-1	31.9b	0.70d	1.65d	11.76b	0.91c	0.23c	0.82c
FD73-49	24.0c	1.35c	2.09b	10.34c	0.48e	0.12f	0.44e
Sante	24.7c	1.50b	1.94c	9.13d	0.98b	0.29b	0.89b
PRI Red	16.3e	3.15a	2.18a	8.38e	1.07a	0.31a	0.97a
FD51-5	18.5d	1.56b	2.11b	8.90d	0.65d	0.13e	0.59d
LSD G (P ≤ 0.05)	1.03	0.120	0.045	0.418	0.02	0.012	0.014
Storage period (SP)							
Week 1	0.0b	0.0b	0.00b	11.24a	0.34b	0.08b	0.32b
Week 3	49.7a	2.97a	3.86a	9.77b	1.32a	0.35a	1.20a
LSD SP (P ≤ 0.05)	0.53	0.069	0.026	0.241	0.015	0.007	0.008
LSD T × G (P ≤ 0.05)	NS	NS	0.090	NS	NS	NS	NS
LSD T × SP (P ≤ 0.05)	1.19	0.13	0.052	0.483	0.017	0.014	0.016
LSD G × SP (P ≤ 0.05)	1.45	0.16	0.063	0.592	0.021	0.018	0.020
LSD T × G × SP (P ≤ 0.05)	NS	NS	0.128	NS	NS	NS	NS

*DP* dormancy period, *SL* sprout length, *WL* weight loss.

*NS* non-significant at *P* ≤ 0.05. Treatment means sharing the same letter are non-significantly different. LSD is the least significant difference.

**Figure 6:**
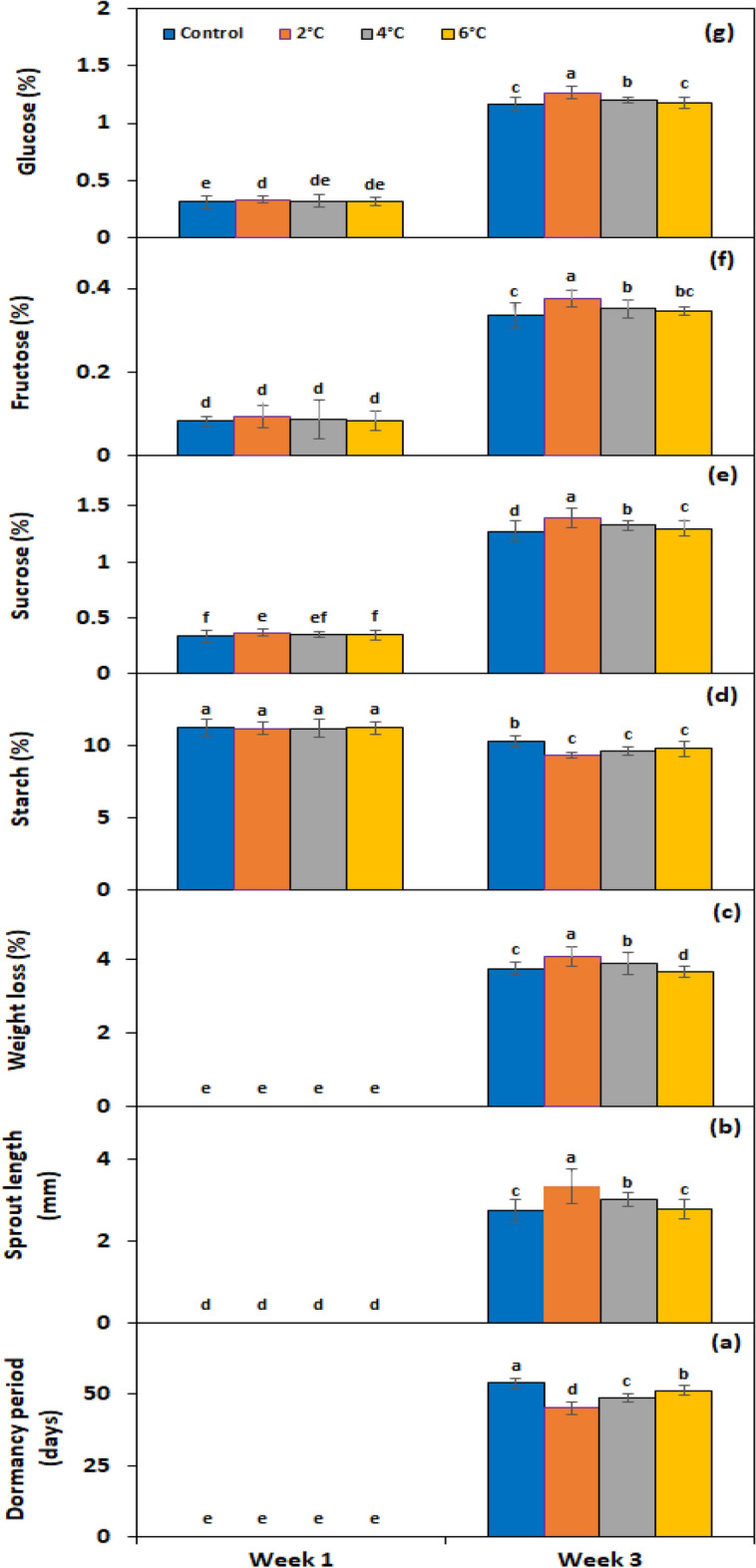
Interactive effect of *temperature* × *storage period* on tuber dormancy period (**a**), sprout length (**b**), weight loss (**c**), starch (**d**), sucrose (**e**), fructose (**f**), and glucose contents (**g**) of six potato genotypes cold stored at low temperature. The treatment means sharing the same letter are non-significant (*P* > 0.05) according to the least significant difference test. The vertical bars represent the standard error of means (n: 3).

**Figure 7:**
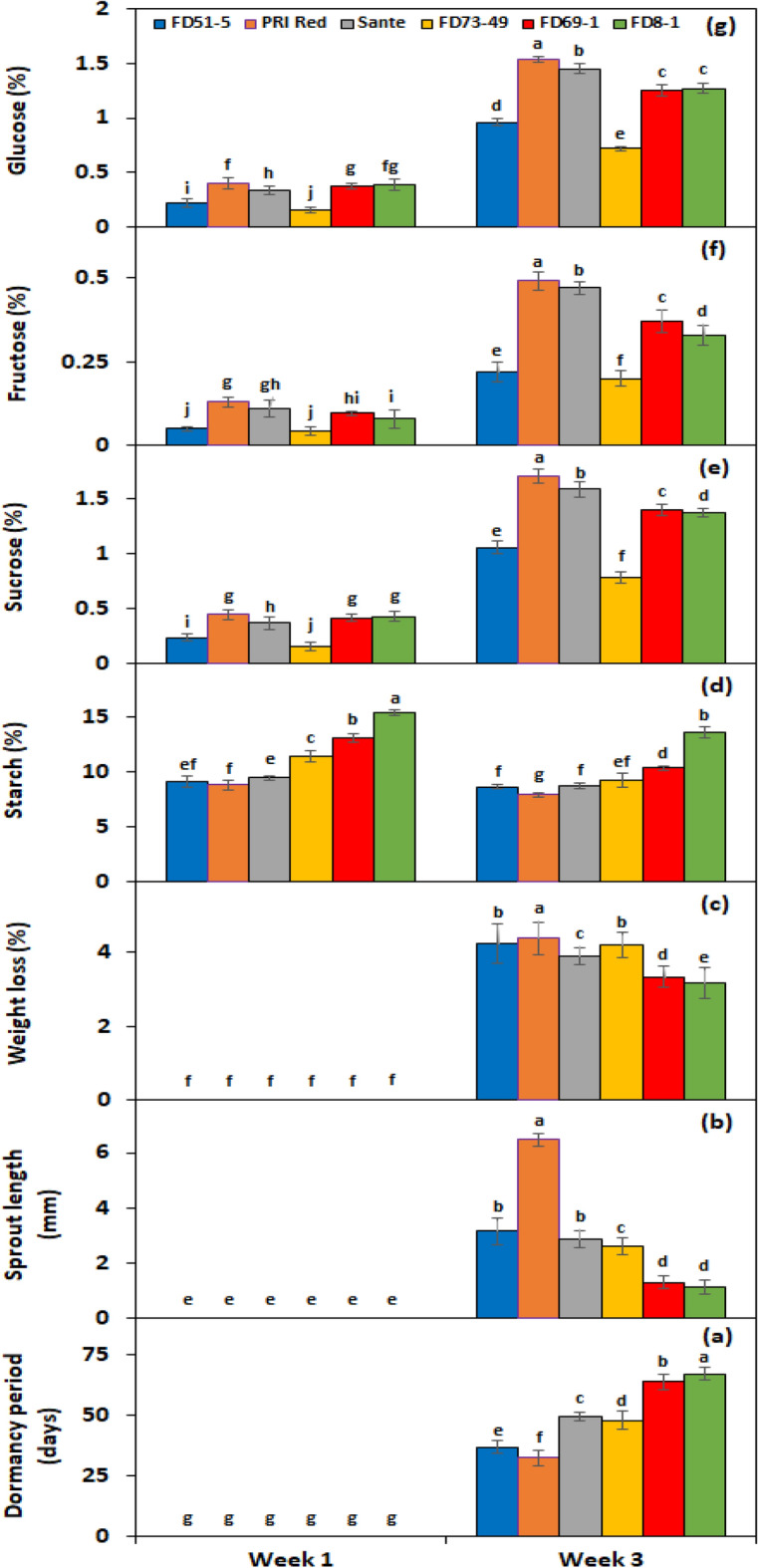
Interactive effect of *genotype* × *storage period* on tuber dormancy period (**a**), sprout length (**b**), weight loss (**c**), starch (**d**), sucrose (**e**), fructose (**f**), and glucose contents (**g**) of six potato genotypes cold stored at low temperature. The treatment means sharing the same letter are non-significant (*P* > 0.05) according to the least significant difference test. The vertical bars represent the standard error of means (n: 3).

Under the *temperature* × *storage period* interaction, the dormancy period was significantly broken earlier by almost 8.7 days compared to the control when tubers were kept at 2 °C (Fig. 6a). The tubers kept at this temperature also showed 18.2% greater sprout length (Fig. 6b) and 7.9% more weight loss (Fig. 6c) than control tubers. Under genotype × storage period interaction, PRI Red advanced rapidly towards dormancy breakage (32.6 days) (Fig. 7a), with the longest sprout at week 3 (6.5 mm) (Fig. 7b) due to the rapid rate of decrease in starch contents and increase in sugar contents, which resulted in a remarkable drop in its weight (4.4%) (Fig. 7c). On the other hand, FD8-1 displayed the longest tuber dormancy period (67.2 days) (Fig. 7a), with shortest sprout length (1.2 mm) (Fig. 7b), and the lowest weight loss (3.2%) (Fig. 7c).

#### Effect on endogenous starch, sucrose, fructose and glucose contents of tuber

The starch contents decreased (10.27%) with the lowest temperature (2 °C) treatment. The sucrose (0.88%), fructose (0.23%), and glucose (0.79%) contents, on the other hand, were enhanced by cold pre-treatment at 2 °C (Table 3). Among genotypes, the maximum starch contents were recorded in FD8-1 (14.51%), while the minimum was in PRI Red (8.38%) (Table 3). Alternatively, sucrose, fructose, and glucose were found to be highest in PRI Red and lowest in FD73-49 (Table 3). There was a weak negative (*r* =  − 0.3) correlation of starch contents with tuber dormancy period and a moderate (*r* =  − 0.5) correlation with sprout length, and weight loss. However, tuber glucose, fructose, and sucrose contents had a strong positive correlation (*r* = 0.8) with dormancy period, sprout length and weight loss. From the first to the third week of storage, starch contents lessened while sucrose, fructose, and glucose contents enhanced (Table 3).

Under *Temperature* × *storage period* interaction, the lowest temperature, i.e*.*, 2 °C, decreased the starch contents by 49.5% (Fig. 6d) and increased the sucrose (Fig. 6e), fructose (Fig. 6f), and glucose (Fig. 6g) contents by 9%, 9.6%, and 9.3% correspondingly. Under the interactive effect of *genotype* × *storage period*, starch levels dropped more quickly in the short-term dormancy genotypes than in the moderate or long-term dormancy genotypes (Fig. 7d). On the other hand, sucrose (Fig. 7e), fructose (Fig. 7f), and glucose (Fig. 7g) contents were developed in small quantities in the tubers of PRI Red during the first week in comparison with the contents during the third week.

### Electric shock of tubers

#### Effect on tuber dormancy period, sprout length and weight loss

The tuber dormancy period, sprout length, and weight loss were significantly affected by the electric current, genotypes, storage periods, and their interactions: *electric current* × *storage period* and *genotype* × *storage period* (Table 4). The tuber weight loss was also affected by *electric current* × *genotype*, and *electric current* × *genotype* × *storage period* (Table 4). The tubers treated with the electric current at 80 V had the shortest dormancy period (20.5 days), which was statistically different from the control. The highest sprout length (1.74 mm) and weight loss (2.11%) were also recorded in the tubers treated with 80 V electric current. Among genotypes, PRI Red exhibited the shortest dormancy period (15.4 days), highest sprout length (3.24 mm), and weight loss (2.14%) while FD8-1 had the longest period (31.4 days), smallest sprout length (0.58 mm), and weight loss (1.70%) (Table 3). As the storage period advanced, tuber dormancy decreased with an increase in sprout length and weight loss.

**Table 4:** Effect of electric current on tuber dormancy period, sprout length and weight loss of six potato genotypes and relative changes occurred in in endogenous starch, fructose, glucose and sucrose contents.

Factors	DP (days)	SL (mm)	WL (%)	Starch (%)	Sucrose (%)	Fructose (%)	Glucose (%)
Electric current (Ec)							
0-V	27.2a	1.37b	1.87e	10.73a	0.81c	0.212c	0.74d
80-V	20.5d	1.74a	2.11a	10.17c	0.90a	0.247a	0.83a
60-V	23.2c	1.44b	2.03b	10.35bc	0.84b	0.221b	0.77b
40-V	23.9c	1.38b	1.97c	10.39bc	0.83b	0.219b	0.77b
20-V	25.1b	1.37b	1.92d	10.58ab	0.82c	0.215c	0.75c
LSD T (P ≤ 0.05)	0.80	0.097	0.027	0.333	0.009	0.004	0.009
Genotype (G)							
FD8-1	31.4a	0.58e	1.70d	14.50a	0.88c	0.20d	0.82c
FD69-1	30.2b	0.65e	1.73c	11.68b	0.89c	0.24c	0.82c
FD73-49	24.2c	1.30d	2.17a	10.37c	0.51e	0.12f	0.46e
Sante	24.1c	1.44c	1.98b	8.97d	1.01b	0.29b	0.92b
PRI Red	15.4e	3.24a	2.14a	8.27e	1.11a	0.32a	1.02a
FD51-5	18.5d	1.57b	2.16a	8.86d	0.64d	0.14e	0.59d
LSD G (P ≤ 0.05)	0.88	0.106	0.029	0.365	0.010	0.005	0.021
Storage period (SP)							
Week 1	0.0b	0.0b	0.00b	11.10a	0.35b	0.08b	0.32b
Week 3	48.0a	2.92a	3.97a	9.79b	1.33a	0.35a	1.23a
LSD SP (P ≤ 0.05)	0.50	0.061	0.017	0.210	0.006	0.002	0.006
LSD Ec × G (P ≤ 0.05)	NS	NS	0.066	NS	NS	NS	NS
LSD Ec × SP (P ≤ 0.05)	1.13	0.13	0.038	0.471	0.013	0.006	0.013
LSD G × SP (P ≤ 0.05)	1.24	0.15	0.042	0.516	0.015	0.007	0.015
LSD Ec × G × SP (P ≤ 0.05)	NS	NS	0.094	NS	NS	NS	NS

*DP* dormancy period, *SL* sprout length, *WL* weight loss.

*NS* non-significant at *P* ≤ 0.05. Treatment means sharing the same letter are non-significantly different. LSD is the least significant difference.

**Figure 8:**
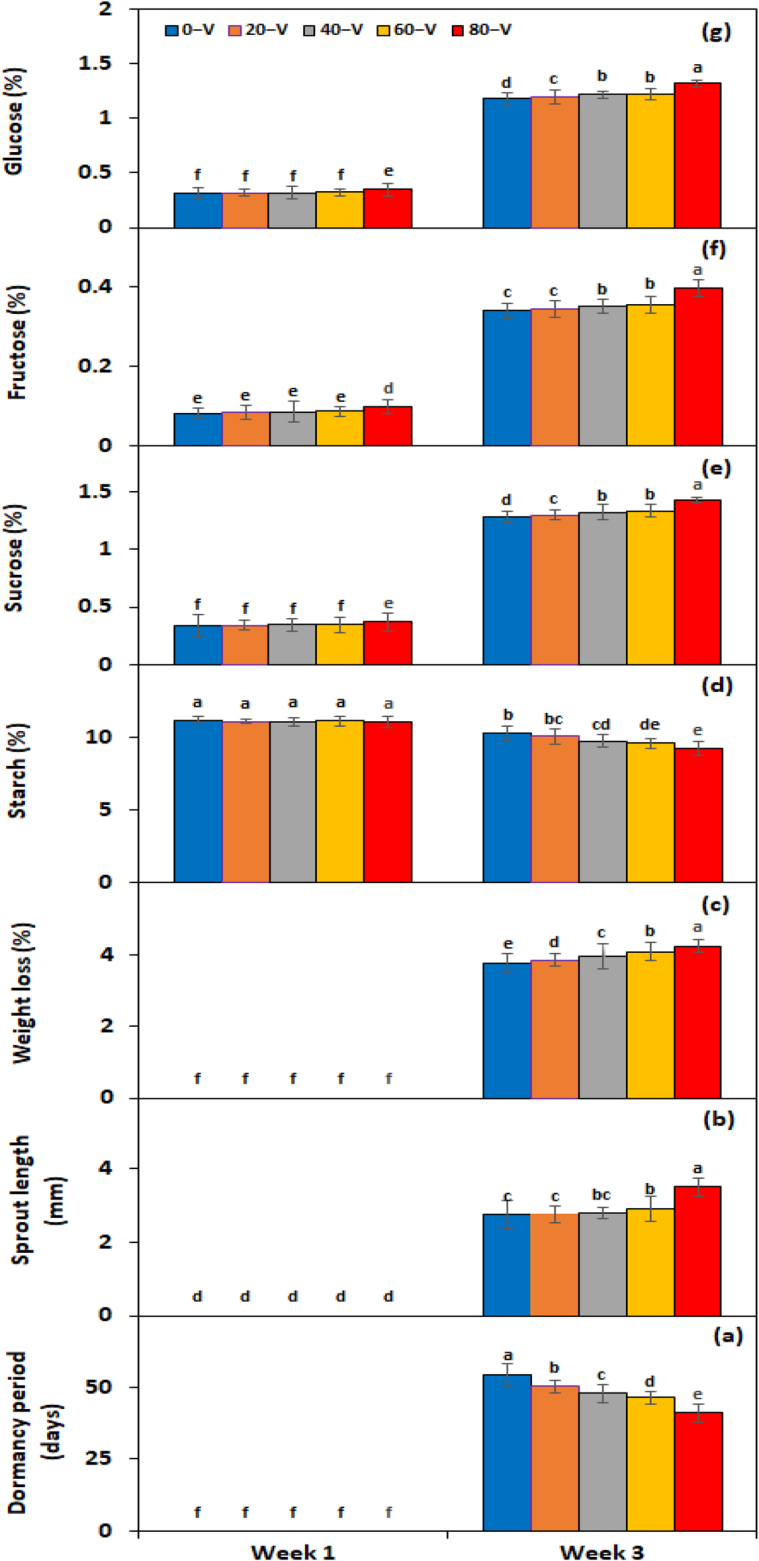
Interactive effect of *electric current* × *storage period* on tuber dormancy period (**a**), sprout length (**b**), weight loss (**c**), starch (**d**), sucrose (**e**), fructose (**f**), and glucose contents (**g**) of six potato genotypes treated with electric current. The treatment means sharing the same letter are non-significant (*P* > 0.05) according to the least significant difference test. The vertical bars represent the standard error of means (n: 3).

**Figure 9:**
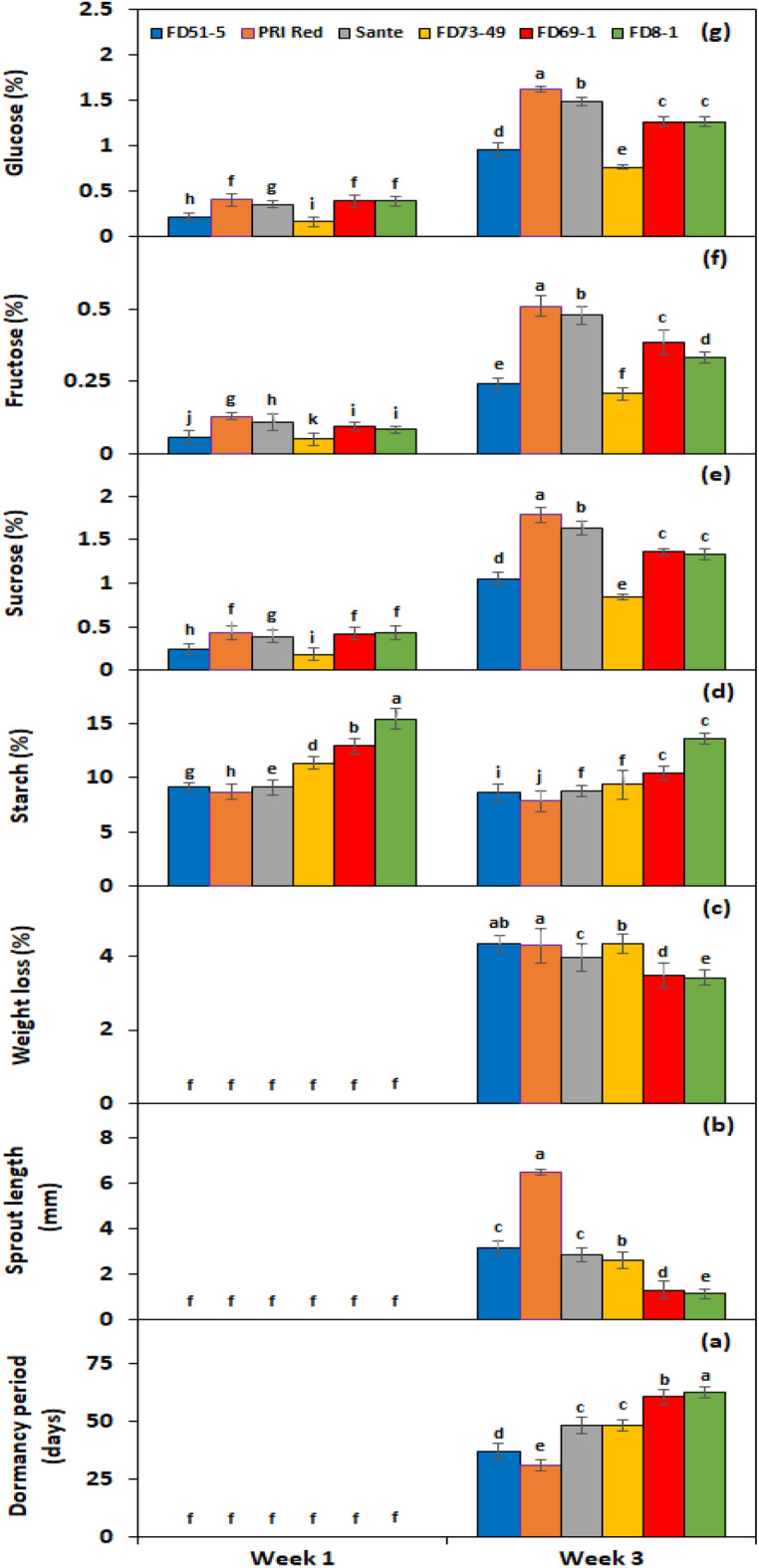
Interactive effect of *genotype* × *storage period* on tuber dormancy period (**a**), sprout length (**b**), weight loss (**c**), starch (**d**), sucrose (**e**), fructose (**f**), and glucose contents (**g**) of six potato genotypes treated with electric current. The treatment means sharing the same letter are non-significant (*P* > 0.05) according to the least significant difference test. The vertical bars represent the standard error of means (n: 3).

Under the *electric current* × *storage period* interaction, the dormancy period was significantly broken almost 13.2 days before in comparison with the control when tubers were treated with 80 V electric current (Fig. 8a). The tubers treated with 80 V electric current also showed 21.2% greater sprout length (Fig. 8b) and 11.5% more weight loss (Fig. 8c) than control tubers. Under *genotype* × *storage period* interaction, PRI Red advanced rapidly towards dormancy breakage (32.6 days) (Fig. 9a), with the longest sprout at week 3 (6.48 mm) (Fig. 9b) due to the rapid rate of decrease in starch contents and increase in sugar contents, which resulted in a remarkable drop in its weight (4.37%) (Fig. 9c). On the other hand, FD8-1 displayed the longest tuber dormancy period (67.3 days) (Fig. 9a), the shortest sprout length (1.15 mm) (Fig. 9b), and the lowest weight loss (3.17%) (Fig. 9c).

#### Effect on endogenous starch, sucrose, fructose and glucose contents of tuber

The starch contents decreased (10.17%) with the highest volt electric current (80 V). On the other hand, electric current applied at 80 V enhanced the contents of sucrose (0.90%), fructose (0.25%), and glucose (0.83%) (Table 4). Among genotypes, the maximum starch contents were recorded in FD8-1 (14.50%), while the minimum was in PRI Red (8.27%) (Table 4). Alternatively, sucrose, fructose, and glucose were found to be highest in PRI Red and lowest in FD73-49 (Table 4). There was a weak negative (*r* =  − 0.25) correlation of starch contents with tuber dormancy period and a moderate (r ≥ 0.45) correlation with sprout length and weight loss. However, tuber glucose, fructose, and sucrose contents had a strong positive correlation (*r* = 0.8) with dormancy period, sprout length, and weight loss. During 3 weeks of storage, starch contents decreased while sucrose, fructose, and glucose contents increased (Table 4).

Under *electric current* × *storage period* interaction, the highest volt electric current (80 V) decreased the starch contents by two folds (Fig. 8d) and increased the sucrose (Fig. 8e), fructose (Fig. 8f), and glucose (Fig. 8g) contents by 11.2%, 13.7%, and 11% correspondingly. Under the interactive effect of genotype × storage period, starch levels declined quicker in the short-term dormancy genotypes than in the moderate or long-term dormancy genotypes (Fig. 9d). On the other hand, sucrose (Fig. 9e), fructose (Fig. 9f) and glucose (Fig. 9g) contents were developed in small quantities in the tubers of PRI Red during first week in comparison with the contents during third week.

### Irradiation of tubers

#### Effect on tuber dormancy period, sprout length and weight loss

γ-Rays, genotypes, storage periods, and their interactions: *irradiation* × *storage period* and *genotype* × *storage period* had a significant (*P* ≤ 0.05) effect on tuber dormancy period, sprout length, and weight loss (Table 5). The tuber weight loss was also affected by *γ-rays* × *genotype* and *γ-rays* × *genotype* × *storage period* (Table 5). The lowest dormancy period (24.5 days) was noted in the tubers treated with the highest dose of radiation (3.5 kGy) (Table 5). The highest sprout length (1.6 mm) and weight loss (2.1%) were also noticed in the tubers exposed to 3.5 kGy γ-rays. Among genotypes, PRI Red exhibited the shortest dormancy period (18.1 days), highest sprout length (3.32 mm), and weight loss (2.10%), whereas FD8-1 took the longest period (34.4 days) to dormancy breakage (Table 5), lowest sprout length (0.52 mm), and weight loss (1.67%). From the first to the third week of storage, their dormancy period decreased with an increase in their sprout length and weight loss.

**Table 5:** Effect of γ-radiations on tuber dormancy period, sprout length and weight loss of six potato genotypes in relation to endogenous changes occurred in starch, sucrose, fructose and glucose contents of tuber.

Factors	DP (days)	SL (mm)	WL (%)	Starch (%)	Sucrose (%)	Fructose (%)	Glucose (%)
Irradiation (I)							
Control	27.8a	1.42b	1.90d	11.24a	0.799c	0.205c	0.720c
3.5 kGy	24.5e	1.60a	2.10a	10.75f	0.856a	0.228a	0.779a
3.0 kGy	26.1d	1.48b	2.02b	10.82e	0.822b	0.215b	0.746b
2.5 kGy	26.3cd	1.47b	1.92c	10.88d	0.815bc	0.211bc	0.739bc
2.0 kGy	26.9bc	1.42b	1.92c	10.95c	0.810bc	0.209bc	0.736bc
1.5 kGy	27.2ab	1.43b	1.90cd	11.08b	0.808bc	0.208c	0.731bcd
1.0 kGy	27.7a	1.42b	1.92cd	11.08b	0.802c	0.206c	0.726cd
LSD I (*P* ≤ 0.05)	**0.73**	**0.086**	**0.025**	**0.007**	**0.015**	**0.006**	**0.016**
Genotype (G)							
FD8-1	34.4a	0.52f.	1.67e	14.77a	0.88c	0.20d	0.81c
FD69-1	34.5a	0.61e	1.88d	12.80b	0.88c	0.23c	0.79c
FD73-49	26.2c	1.31d	2.11a	11.01c	0.48e	0.12f	0.43e
Sante	27.5b	1.43c	1.92c	10.04d	0.98b	0.28b	0.88b
PRI Red	18.1e	3.32a	2.10a	8.29f	1.04a	0.30a	0.95a
FD51-5	19.2d	1.60b	2.05b	8.96e	0.60d	0.13e	0.55d
LSD G (*P* ≤ 0.05)	**0.67**	**0.080**	**0.023**	**0.007**	**0.014**	**0.005**	**0.015**
Storage period (SP)							
Week 1	0.0b	0.00b	0.00b	12.05a	0.34b	0.08b	0.31b
Week 3	53.3a	2.93a	3.92a	9.90b	1.28a	0.34a	1.16a
LSD SP (*P* ≤ 0.05)	**0.39**	**0.046**	**0.012**	**0.004**	**0.008**	**0.003**	**0.008**
LSD I × G (*P* ≤ 0.05)	NS	NS	0.060	0.019	NS	NS	NS
LSD I × SP (*P* ≤ 0.05)	1.04	0.122	0.034	0.011	0.022	0.008	0.023
LSD G × SP (*P* ≤ 0.05)	0.96	0.113	0.032	0.010	0.021	0.008	0.021
LSD I × G × SP (*P* ≤ 0.05)	NS	NS	0.085	0.027	NS	NS	NS

Significant values are in [bold].

*NS* non-significant at *P* ≤ 0.05. Treatment means sharing the same letter are non-significantly different. LSD is the least significant difference.

*DP* dormancy period, *SL* sprout length, *WL* weight loss.

**Figure 10:**
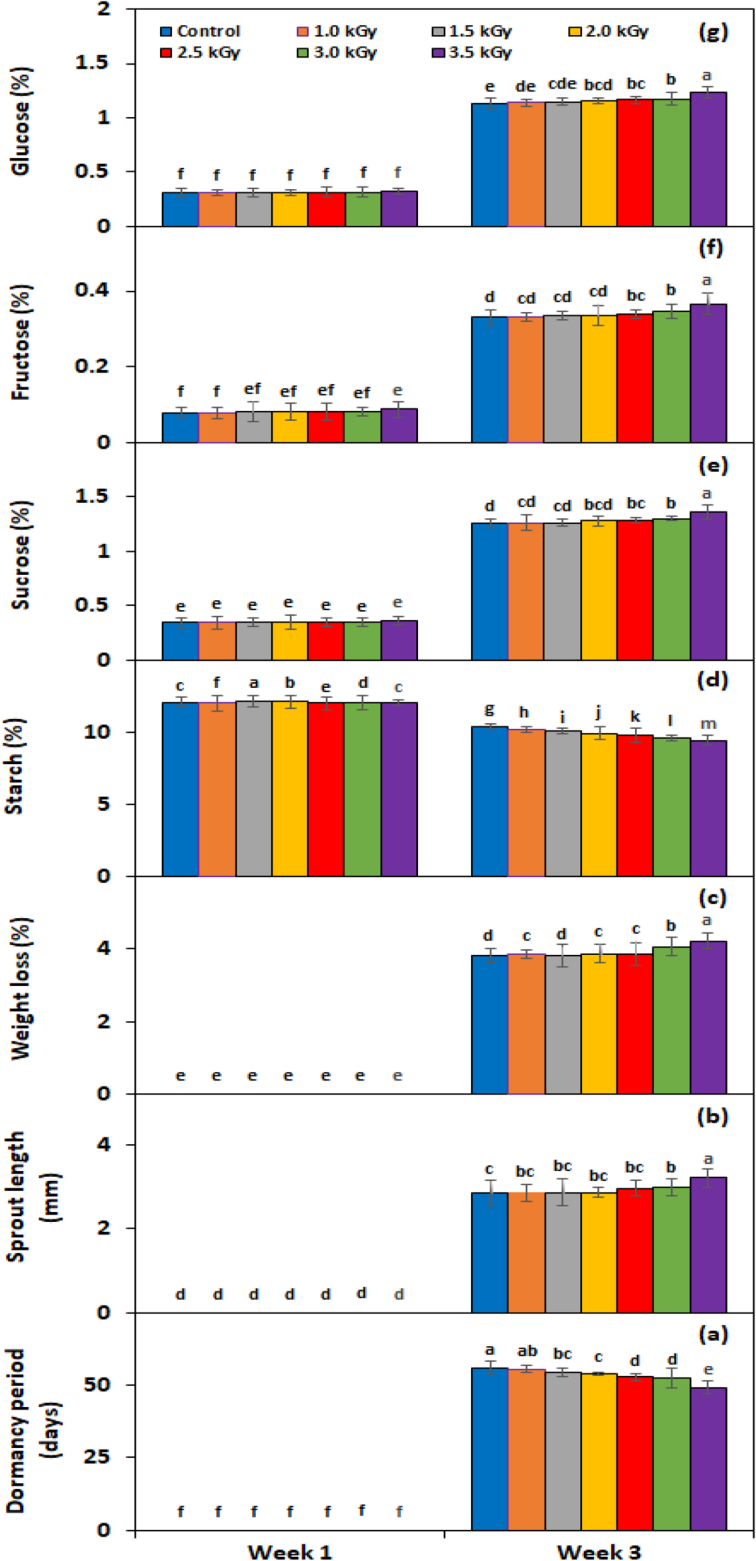
Interactive effect of *irradiation* × *storage period* on tuber dormancy period (**a**), sprout length (**b**), weight loss (**c**), starch (**d**), sucrose (**e**), fructose (**f**), and glucose contents (**g**) of six potato genotypes exposed to γ-rays. The treatment means sharing the same letter are non-significant (*P* > 0.05) according to the least significant difference test. The vertical bars represent the standard error of means (n: 3).

**Figure 11:**
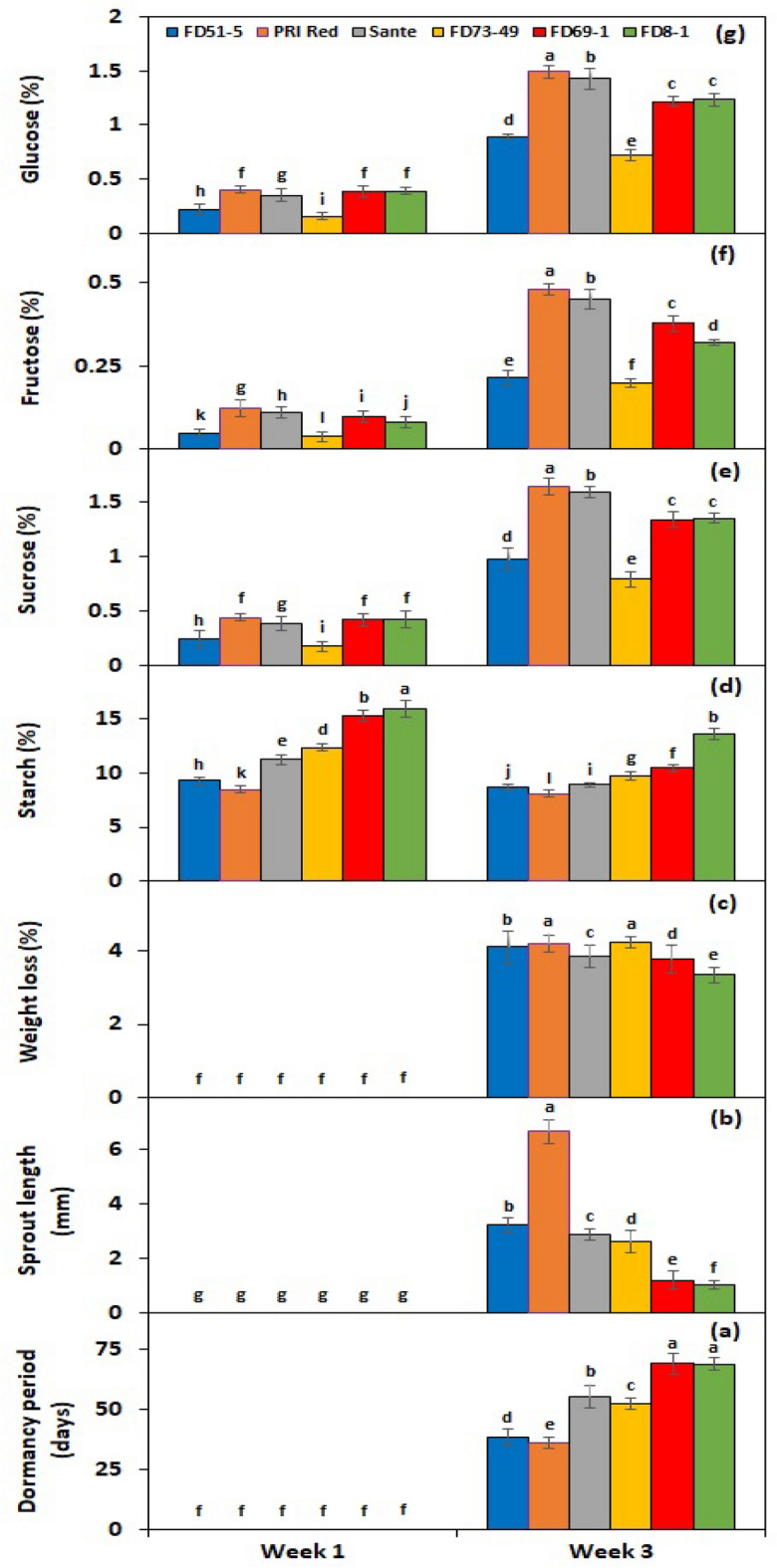
Interactive effect of *genotype* × *storage period* on tuber dormancy period (**a**), sprout length (**b**), weight loss (**c**), starch (**d**), sucrose (**e**), fructose (**f**), and glucose contents (**g**) of six potato genotypes exposed to γ-rays. The treatment means sharing the same letter are non-significant (*P* > 0.05) according to the least significant difference test. The vertical bars represent the standard error of means (n: 3).

Under the *irradiation* × *storage period* interaction, the dormancy period was significantly broken almost 6.5 days before in comparison with the control when tubers were treated with 3.5 kGy γ-rays (Fig. 10a). The tubers treated with 3.5 kGy γ-rays also gave 11.3% greater sprout length (Fig. 10b) and 9.5% more weight loss (Fig. 10c) than control tubers. Under genotype × storage period interaction, PRI Red advanced rapidly towards dormancy breakage (36.2 days) (Fig. 11a), with the longest sprout at week 3 (6.64 mm) (Fig. 11b) due to the rapid rate of decrease in starch contents and increase in sugar contents, which resulted in a remarkable drop in its weight (4.2%) (Fig. 11c). On the other hand, FD8-1 displayed the longest tuber dormancy period (68.9 days) (Fig. 11a), the shortest sprout length (1.05 mm) (Fig. 11b), and the lowest weight loss (3.34%) (Fig. 11c).

#### Effect on endogenous starch, sucrose, fructose and glucose contents of tuber

The starch, sucrose, fructose, and glucose contents in the experimental tubers were significantly affected by γ-rays, genotypes, storage periods, and the two-way interactions of *irradiation* × *storage period*, and *genotype* × *storage period* (Table 5). The contents of starch, sucrose, fructose, and glucose for γ-rays, genotypes, and storage periods are given in Table 5. The starch contents were significantly decreased (10.75%) in the tubers treated with 3.5 kGy γ-rays (Table 5). The sucrose (0.856%), fructose (0.228%), and glucose (0.779%) contents of tubers, on the other hand, were found highest with 3.5 kGy γ-rays. Among genotypes, FD69-1 and FD8-1 exhibited the highest levels of starch, while PRI depicted the lowest level (Table 5). Alternatively, sucrose, fructose and glucose were found to be highest in PRI Red and lowest in FD73-49 (Table 5). There was a weak negative (*r* =  − 0.3) correlation of starch contents with tuber dormancy period and weight loss or a moderate (*r* =  − 0.45) correlation with sprout length. However, tuber glucose, fructose, and sucrose contents had a strong positive correlation (*r* = 0.8) with dormancy period, sprout length, and weight loss. From the first to the third week of storage, starch contents declined while sucrose, fructose, and glucose contents elevated (Table 5).

Under *irradiation* × *storage period* interaction, the highest dose γ-rays (3.5 kGy) decreased the starch contents by 36.1% (Fig. 10d) and increased the sucrose (Fig. 10e), fructose (Fig. 10f), and glucose (Fig. 10g) contents by 8.3%, 9.7%, and 9.6% correspondingly. Under *genotype* × *storage period* interaction, starch contents reduced more quickly in the short-term dormancy genotypes than in the moderate or long-term dormancy genotypes (Fig. 11d). On the other hand, sucrose (Fig. 11e), fructose (Fig. 11f), and glucose (Fig. 11g) contents were developed in small quantities in the tubers of PRI Red during the first week in comparison with the contents during the third week.

## Discussion

Pakistan's seed potato industry demands genotypes with variable tuber dormancy length to accommodate the growers' single or multiple cropping schedules^5,10^. Moreover, the use of dormancy breaking techniques provides even more flexibility in raising consecutive crops^6^. The use of chemicals to shorten tuber dormancy, such as thiourea, bromoethane, and rindite, has been extensively investigated, but these substances are hazardous to both humans and the environment^25^. It is therefore imperative to characterize the germplasm in order of their dormancy period, which either directly or indirectly controls the maturation time^39^ and keeping quality^40^ of the potato crop, and devise an effective, safe, and environmentally acceptable technique for breaking seed-tuber dormancy to enable single or multiple cropping systems^6^.

Dormancy (G_0_ phase) is induced in the tubers from the inhibition of the flow of reducing sugars, such as fructose and glucose, from the G_1_-to-S and G_2_-to-M transitions during the cell cycle, as a result no cell development takes place^28^. The similar findings were obtained in our study as the control tubers of all studied genotypes showed a growth arrest after 1 week of treatment in term of no dormancy breakage. This might be attributed to the less/ no production of reducing (fructose and glucose) and/or non-reducing (sucrose) sugars (Figs. 2a, 4a, 6a, 8a, 10a). After week 3, short-term and moderate-term dormancy genotypes initiated sprouting which might be due to production of fructose and glucose as a result of starch conversion. In fact, dormancy induction is evolved by the tubers as a protective mechanism against unfavorable conditions^15^. This fact can be proved from the findings of Suttle^41^ that the period of dormancy is inherently longer in wild genotypes in comparison to genotypes developed through modern breeding. The group Phureja of *Solanum tuberosum* is exempted from this fact, as their tubers have a short or no dormancy^42^.

The application of cytokinin exogenously induces changes in the transport of endogenous nutrients and create sink regions to attract photosynthates. It has been suggested that the nutritional sink effect of benzylaminopurine (BAP), which is crucial for maintaining the G_1_-S and G_2_-M transitions in the plant cell, may be the cause of the shortening dormancy period. While the application of GA_3_ causes the starch breakdown in tubers, since they are responsible for producing enzymes like amylase that assist in the conversion of starch into sugars through adjustments in intracellular compartmentation^43^. So, combining the application of both cytokinin and gibberellin will be more effective since cytokinins terminate dormancy and gibberellins will increase sprout growth. In this study, BAP performed best at 60 mg L^−1^ in lowering dormancy period (18.4 days) while GA_3_ at 20 mg L^−1^ for sprout length (2.05 mm) (Table 1). The combination of both BAP and GA_3_ at their best levels showed the synergistic effect by quickly breaking the dormancy (18 days) and producing longer sprouts (2.08 mm) as compared to their induvial applications (Table 2). The significant decrease in starch content in tubers treated with 60 mg L^−1^ BAP and 20 mg L^−1^ GA_3_ suggests that these treatments might have triggered metabolic processes leading to starch degradation. BAP is a growth-promoting hormone that may have induced starch mobilization in the tubers. While GA_3_ can influence the activity of enzymes responsible for starch breakdown. Similar results were obtained by Claassens and Vreugdenhil^43^ and Njogu et al.^44^ who compared the individual and combined effect of BAP and GA_3_ on potato tuber dormancy and sprout length and found the decisive role of cytokinin in terminating dormancy and that of GA_3_ in encouraging sprout length. Little soluble sugars are present in the resting buds, but as soon sprouting begins, α- and β-amylases emerge in the tubers which starts converting starch into soluble sugars in the sub-eye regions, to maintain the sprout growth^45^. In fact, the soaking of potato tubers in GA_3_ solution increases the endogenous concentrations of GAs, which affects the production of amylase, that in turn affects the starch breakdown and accelerates sprout outgrowth. Elevation in the contents of soluble sugars at dormancy break suggested that sucrose release into buds is essential for initiation of sprouting. Low sucrose levels in the buds may act as a signal to regulate parenchymal starch transport^46^. There is a noticeable decrease in starch contents, which can be related to the buildup of soluble sugars at sprouting^47^. As in our study, there were comparatively higher starch and lower sucrose, fructose and glucose contents before commencement of sprouting. After third week of PGRs, as sprouting started, starch contents decreased and sucrose, fructose and glucose contents increased (Tables 1, 2). The sugars developed at commencement of sprouting might be utilized by the growing sprouts as earlier reported by Haider et al.^10^. With the growth of sprouts, weight loss increases in tubers as a strong correlation exists between weight loss and sprout length and numbers. In this study the combined application of optimized levels of BAP and GA_3_ resulted in quicker dormancy break, more sprout growth and more weight loss as earlier indicated by Pande et al.^15^ Further investigation is needed to elucidate the specific enzymatic pathways and physiological mechanisms involved in starch degradation under these treatments.

In potatoes, low or high temperature shock shortens the tuber dormancy^48^. In this study, the effect of cold pre-treatment on tuber dormancy period and sprout length was found substantial when compared with the control. The findings are in line with those of Muthoni et al.^49^ who discovered that a 2 °C cold pre-treatment of tubers reduced the length of dormancy by 14 days in long-term dormancy cultivars. The findings of the present study disagree with the earlier reports which showed that cold pre-treatment has no significant influence on dormancy period of short-term dormancy cultivars^34,50^. The shortening of tuber dormancy may be attributed to disruption of membrane by low temperature which resulted in electrolyte leakage and subcellular compartmentation.

The effect of electric current on tuber dormancy, sprout length and weight loss in relation to changes in endogenous starch and sugar contents has never been documented before. The significant differences observed among genotypes for dormancy period and sprout length under the effect of electric current are consistent with the findings of Haider et al.^10^ and Kocacaliskan^32^ who noted a decrease in dormancy days and increase in sprout length as storage time advanced. Although, Kocacaliskan^32^ did not find any significant effect of interactions. The increase in sprouting might be due to stimulating effect of electric current on GAs synthesis, which in turn, enhanced the starch break down into sugars through developing α- and β-amylases; hence caused a quick sprout outgrowth^51^. Electric current application promoted the movement of food reserves from parenchyma tissues to the eyes of the tuber which are used by the sprout for further development.

There is no report to date to shorten tuber dormancy through irradiation although a lot of material has been published on extension of tuber dormancy. γ-Rays are the most disruptive form of electromagnetic radiations^52^. Higher doses of γ-rays can enhance the sugar contents through disrupting the hormonal levels in the tubers. As a result, both reducing and non-reducing sugars start developing. Reducing sugars are utilized by the growing sprout. However, the lower doses of γ-rays have no significant influence on dormancy breakage. According to the present investigation, there were significant differences in starch, sucrose, fructose, and glucose contents between genotypes and storage times in response to the highest dose (3.5 kGy).

## Conclusions

Managing potato tuber dormancy in order to optimise the seed storage and to ensure its availability round the year has now become essential. The potato varieties cultivated in Pakistan typically retain a dormancy period of 2–3 months following harvest, preventing cultivation of spring crop after autumn harvest and autumn crop after summer harvest. In order to enable multiple cropping, Pakistan would need to produce varieties with dormancy of approximately less than two months rather than relying on autumn–autumn seed storage practices. Therefore, based on the time difference between crops, growers may prefer genotypes with a wide range of dormancy. The application of several dormancy-breaking techniques (chemicals, cold pre-treatment, electric current, and radiations) on tubers increases planting flexibility for a succeeding crop. The fastest dormancy breakage and sprout development were obtained when tubers were treated with PGRs, which involved soaking them in solutions containing 60 mg L^−1^ BAP and 20 mg L^−1^ GA_3_. This method is only practically feasible when potatoes are mechanically cut to expose their parenchyma tissues to the PGRs' solutions. The exact timing for dormancy breaking may vary based on factors such as the desired sprout length, the specific potato variety, and regional climatic conditions. Furthermore, the role of endogenous starch during dormancy progression clearly demonstrates that starch content initially peaked and then gradually decreased. Alternatively, reducing (glucose and fructose) and non-reducing (sucrose) sugars were lowest at the start of dormancy and increased with time. In further work, the authors recommend more research on exploring the genetic basis of dormancy duration in potato genotypes and identifying specific genes or markers associated with dormancy break and sprout length.

### Supplementary Information

Supplementary Information.

## Data Availability

All data generated or analyzed during this study are included in this published article and may also be requested from the corresponding authors.
