# Supplementary material for: Postharvest starch and sugars adjustment in potato tubers of wide-ranging dormancy genotypes subjected to various sprout forcing techniques
Source: Sci Rep. 2023 Sep 8;13:14845. doi: 10.1038/s41598-023-37711-y (PMC10491617; doi:10.1038/s41598-023-37711-y)
Supplement: Supplementary file 1 — Supplementary Information. [file 41598_2023_37711_MOESM1_ESM.docx]

| **No.** | **Genotype** | **Characteristics** | **Average yield** | **Parentage** | **Date of release** |
| --- | --- | --- | --- | --- | --- |
| 1 | PRI Red | Red skinned, late maturing, frost tolerant, oblong. | 30-ton ha^-1^ | FD 44-24 × FD 12-24 | 2013 |
| 2 | FD73-49 | Red skinned, late maturing, oval. | 35-ton ha^-1^ | FD 73-44 × PRI-Red | - |
| 3 | FD8-1 | Red skinned, late maturing, oblong. | 27-ton ha^-1^ | PRI-RED × SH-5 | - |
| 4 | FD51-5 | White skinned, frost tolerant, round oval. | 30-ton ha^-1^ | Sadaf × FD 35-36 | - |
| 5 | Sante | White skinned, Oval to round-oval | 25-ton ha^-1^ | [SVP Y 66 13 636 × SVP AM 66 42](https://www.europotato.org/varieties/view/Sante-E#/) | 1983 |
| 6 | FD69-1 | White skinned, early bulking, round oval. | 27-ton ha-1 | Sialkot Sufaid × FD 3-15 | - |

**Table 1.** Characteristics, yield, parentage, and release information of selected genotypes.
